# Effect of* Boswellia Thurifera* Gum Methanol Extract on Cytotoxicity and *P53* Gene Expression in Human Breast Cancer Cell Line 

**Published:** 2014

**Authors:** Nasrin Yazdanpanahi, Mandana Behbahani, Afsaneh Yektaeian

**Affiliations:** a *Department of Biochemistry, Falavarjan Branch, Islamic Azad University, Isfahan, Iran.*; b *Department of Biotechnology, Faculty of Advanced Sciences and Technologies, University of Isfahan, Iran, Isfahan.*

**Keywords:** Boswellia, Breast cancer, P53 gene, MTT assay, Real time PCR

## Abstract

*Boswellia* has been widely used in traditional medicine for the treatment of different diseases such as cancer in Iran. The aim of this study was to evaluate the effect of the gum methanol extract of *Boswellia thurifera* on the viability and *P53* gene expression of cultured breast cancer cells. The gum methanol extract was obtained in various concentrations using the maceration method. Normal (HEK-293) and cancer (MDA-MB-231) human cells were cultured and treated with various concentrations of the extract. Then MTT assay was used for the study of cytotoxic effect of the extract and real time PCR method was also applied for the investigation of *P53* gene expression in cancer cells. The IC_50_ of the extract against cancer cells was 80 µg/mL and had less cytotoxic effect in normal cells. The effect of the extract was dose dependent. Induction of *P53 *expression by extract was also significantly more in treated cancer cells than untreated cells. This inductive effect in cells was higher after 12 h treatment than it was after 6 h. The results of the current study show that gum methanol extract of *Boswellia thurifera* has probably anti-cancer effects and could induce *P53* gene transcription and toxicity in the cultured breast cancer cell line. The increase of *P53 *gene specific mRNA may be a mechanism of gum methanol extract induced cytotoxicity. However, for a definitive conclusion, further studies on other cell lines as well as animal models and subsequent clinical studies are warranted.

## Introduction

*Boswellia* has been reported traditionally to have beneficial effects like antitumor, analgesia, antiinflammation, antirheumatism, improving intelligence, *etc* (1).

Many pharmacological investigations are performed to detect new drugs for the treatment of diseases such as cancer (2). The weak side-effects and the low cost of medicinal plants in comparison with synthetic drugs have made them valuable products in therapeutic consumptions. Therefore, interest in the study of plants has had a considerable augmentation in recent years. Today there is a growing use of complementary and alternative medicines (CAMs) in different countries, especially developing countries like Iran (3).

The understanding of the mechanisms by which medicinal plant extracts alter the growth, metabolism and viability of cancer cells is needed for cancer therapy (4). 

As cancer cells have unique properties, they are particular targets for various fields of cancer research such as cancer treatment. Undoubtedly, traditional medicine has a major role in better cancer management. 

*P53* is an important tumour suppressor gene and its mutations are among the most frequent genetic alterations detected in different kinds of human cancers. P53 protein prevents the proliferation of abnormal cells, thus inhibiting neoplastic development. Molecular pathological study of the structure and expression of the constituents of the p53 pathway is probably valuable in the diagnosis, prognosis and treatment of cancers such as breast cancer (5).

Different species of* Boswellia *have had various medical uses for many years. They have been applied for treatment of different disorders such as cancer, respiratory diseases, digestive problems, osteoarthritis, *etc *(1, 6-11). 

Geographical conditions create diversity in the gum resins of plants like *Boswellia* (12, 13). So far several investigations have been performed related to the anti-cancer activities of the gum extract of the *Boswellia* species (14-18), but the effect of *Boswellia thurifera* on MDA-MB-231 cells is not known. 

## Experimental

The current search was approved by the Institutional Review Boards of Islamic Azad University of Falavarjan Branch.

*Plant collection*

The aerial part and gum resin of *Boswellia thurifera* were collected from khoszestan. The specimen was identified by Natural Resources Research Center in Isfahan, Iran, in July 2011. The gum was crushed into very small pieces with mortar and pestle, then two hundred and fifty grams of it was placed in a stopped conical flask and macerated with 500 mL methanol (Merck, Germany) for 72 h. The acquired solvent was filtered, concentrated in vacuum at 45 °C to condense and then dried using Freeze Drier (Zirbus, Germany). The crude extract was stored in a well-closed container, protected from light and kept at 4 °C. 

*Cell lines and culture medium*

Two human cell lines including breast cancer cells (MDA-MB-231) and normal human embryonic kidney cells (HEK-293) were acquired from the Pasture Institute cell bank in Tehran (IRAN). MDA-MB-231 and HEK 293 cells were cultured in RPMI-1640 and DMEM, respectively and supplemented with 10% (v/v) Fetal Calf Serum (FCS) (Gibco), 100 U mL^-1 ^penicillin and 100 µgmL^-1^ streptomycin, 2 mM L^-^ Glutamine (Gibco) and 1 mM sodium pyruvate (Gibco). 

*Cytotoxicity assay*

The degree of cytotoxic activity of the extract against breast cancer and normal cells was determined using 3- (4,5-dimethylthiazol-2-yl)-2,5-diphenyl tetrazolium bromide (MTT) assay (19). Two hundred µl of the cell suspension (with a density of approximately 5×10^4^ cells/mL) was transformed to a 96-well plate and incubated for 24 h at 37 °C. DMSO (Dimethyl sulfoxide) was used for preparing of different concentrations of extract. The cells were then treated with extract (with concentrations of 3.5, 7.5, 15.5, 31, 62.5, 125, 250 µg/mL) and incubated for 48 h. Then, 25 μL of the MTT solution (5 mg/mL) was added to each well, and the plate was incubated for 2 h. Finally, the cell culture medium of each well was dicarded and 100 µL DMSO was added to solve formazan crystals. Finally, a micro plate spectrophotometer (Awareness Technology Inc., stat fax 2100) was applied to determine absorbance at 560 nm**.** The percentage of cell viability based on the control (untreated) was estimated according to the following formula: OD of treated cells/OD of untreated cells×100. The survival curves of each cell line were established based on different concentrations of extract after the specified period.

*Quantitative real time polymerase chain reaction*

The expression level of *p53*, a widely established apoptotic-related gene, was analyzed using real time PCR assay. MDA-MB-231 cells were treated with IC_50_ concentration of gum methanol extract (80 µg/mL) during 6 and 12 h periods. Total cellular RNA was extracted from the treated and untreated cells using the TriPure Isolation Reagent (Roche, USA) based on the manufacturer’s instructions. RNA quantification was performed by spectrophotometer (UNICO 2100, USA) using routine procedures. RNA isolates with 260/280 nm absorption ratio > 1.8 were subjected to study. 

Real time PCR was carried out to quantify the amount of mRNA in the treated and untreated cancer cells. A PCR reaction mixture of 50 μL containing 5 μL of ddH_2_O, 25 μL of Taq Man, Universal Master Mix, 5 μL of forward primer, 5 μL of reverse primer, 5 μL of labeled probe (FAM/MGB and or JOE/TAMRA), 1 µL of reverse transcriptase, 2 µL of random hexamer and 2 µL of purified RNA were used. Two pairs of primers were separately applied: one pair to amplify the *p53* gene, the other for the endogenous control gene, *GAPDH*. Primers and probes were selected according to previous study (20) and purchased from metabion incorporation (Table 1).

**Table 1:** Primers and probe of *P53* and *GAPDH*, used for real time analysis

**Gene**	**Sequence**
*P53*	Forward primer: 5′-AGAGTCTATAGGCCCACCCC-3′ Reverse primer: 5′-GCTCGACGCTAGGATCTGAC-3′ Probe: 5′-FAM-TTGGGCAGTGCTCGCT-MGB-3′
*GAPDH*	Forward primer: 5′-CATGGGGAAGGTGAAGGTCGGA-3′ Reverse primer: 5′-TTGGCTCCCCCCTGCAAATGAG-3′ Probe:5′-JOE-CCGACTCTTGCCCTTCGAAC-TAMRA-3′

The RT reaction was started by incubation at 50 °C for 45 min for cDNA synthesis and followed by real time PCR amplification cycles (95 °C for 10 sec and 60 °C for 60 sec, 40 cycles) in a Rotor-Gene 3000 (Corbett Robotics, Australia). A negative control was also used in each run to access specificity of primers and possible contamination. 

*Statistical analysis*

The data was expressed as mean± standard deviation of mean (SD) from three individual tests performed at three different times (with three different repetitions of one test).

SPSS software, one-way analysis of variance (ANOVA) and Post Hoc tests were applied for data analysis. P < 0.05 data were considered statistically significant.

## Results

*MTT assay results*

**Figure 1:**
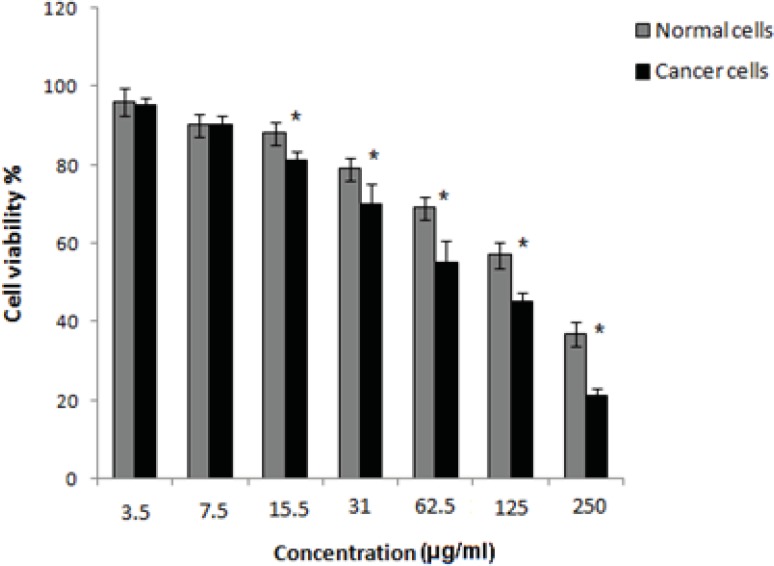
Cell viability assay of normal cells and MDA-MB-231 cancer cells 48 h after treatment *with* *Boswellia thurifera* gum methanol extract. The data was expressed as the mean±SD from 3 independent

The results showed the cytotoxic activity of the gum methanol extract at differnet concentrations. The mean viability percentage after treatment with extract was significantly more in normal cells in comparison with breast cancer cells at a concentration of 15.5 µg/mL and higher. The IC_50_ value was considered as the concentration of the extract that caused a 50% decrease in cell viability relative to the negative control which was constituted by cell culture and DMSO without extract.The IC_50_ of gum methanol extract on cancer and normal cells was 80 and 175 µg/mL, respectively. There was less cytotoxic effect on normal cells for the IC50 concentration of cancer cells (Figure 1). 

*Real time PCR data*

**Figure 2:**
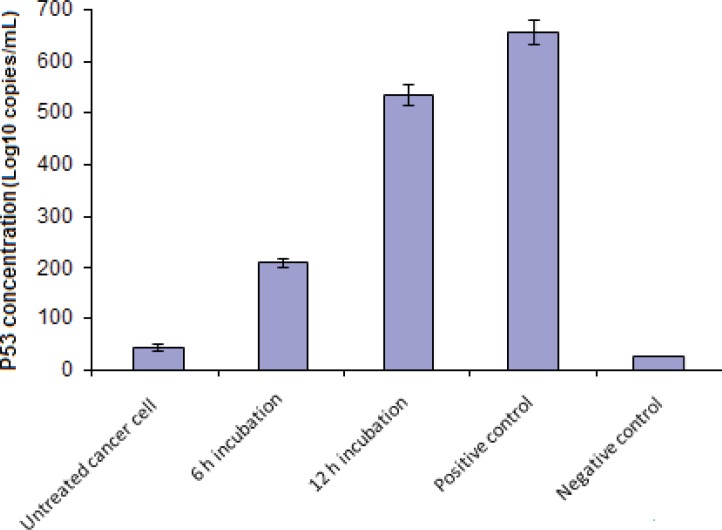
The logarithmic plot of *P53* cDNA concentration in various samples. The data was expressed as the mean ± SD from 3 independent experiments

The real time PCR showed a sudden increase in the *P53* mRNA level in (MDA-MB-231) cells treated with gum methanol extract in comparison with untreated cells (Figure 2). The inductive effect in cells was higher after 12 h treatment than it was after 6 h. These results suggest that the gum extract of *Boswellia thurifera* can augment *P53* gene expression in the cultured breast cancer cell line. The increase of *P53* gene specific RNA probably suggests a mechanism of extract- induced cell death and cytotoxicity.

The logarithmic plot of *P53* cDNA concentration among different samples (breast cancer cells treated with IC_50_ concentration (80 µg/mL) of gum methanol extract during 6 and 12 h periods, untreated breast cancer cells, positive and negative controls) was prepared using real-time PCR analysis (Figure 2)*.*

## Discussion

The extract compositions and properties of *Boswellia *are dependent on species, soil and climate, (12, 13). Investigation of different extracts of *Boswellia *species is usefull for evaluation of medicinal and pharmacological efficacies of these plants.

In the current study the anti-cancer effect of the gum methanol extract of *Boswellia thurifera* on MDA-MB-231 was studied. Since several investigations have shown that the polar (such as methanol) extracts of plants have stronger anti-cancer effects than nonpolar extracts (21-23), the decision was made to evaluate the cytotoxic activity of gum methanol extract in the present research. Evidence that the* Boswellia *species exhibit anti-cancer activity has been found in several previous studies (8-10, 14-18). The *in-vitro* cytotoxic effects of these plants on various cell lines has been reported in other investigation but the anti-cancer effect of* Boswellia thurifera* on MDA-MB-231 cell line has not yet been studied. In one investigation boswellic acids indicated anti-cancer effect against MCF-7 breast cancer cells (15). Essential oil of the gum resin of *Boswellia sacra* induced *in-vitro* death and apoptosis of breast tumor cells (16). In another study, frankincense oil derived from *Boswellia carteri* supressed bladder cancer cells (14). The inhibition effect of *Boswellia* species on other cancer cells such as breast cancer brain metastases and leukemia cells has also been indicated (17, 18).

*In-vitro *studies of the effect of herbal extracts on various cancer and normal cell lines are important for the identification of molecular mechanisms involved in cancer and designing new treatment strategies and drugs for this disease. 

The results as presented here show that gum methanol extract from *Boswellia thurifera* has a dose dependent cytotoxic effect on the cultured MDA-MB-231 cell line. The data also suggests the increase of *P53* gene transcription in these cells in the presence of gum methanol extract. Change of *P53* expression was time dependent and may be a mechanism of cell death in response to the gum extract. 

## Conclusion

In the present study gum methanol extract of* Boswellia thurifera* suppressed survival and induced cytotoxicity and *P53* gene expression in cultured breast cancer cells. Thus, it may be a good candidate for use as an inhibitor of the growth of cancer cells *in-vivo* and the treatment of breast cancer. However, for a definitive conclusion, further *in-vivo* and *in-vitro* studies on other cell lines, animal models and subsequent clinical studies are warranted.

The result also supports the fact that a large number of anti-cancer compounds can be from polar agents (21-23). 
